# Advancing the Study of Subjective Age: More Seriously Considering Gender

**DOI:** 10.1093/geroni/igaa057.1825

**Published:** 2020-12-16

**Authors:** Shelbie Turner, Karen Hooker, Toni Calasanti

## Abstract

Socially-cued age expectations inform people’s subjective age - that is, how old they feel relative to their chronological age. Age-graded expectations are widely considered to be gendered, yet gender has not often been empirically examined as the scholarship on subjective age has developed. Because subjective age shapes the experiences a person has becoming and being an older adult, and is an important correlate of later life health, more seriously considering gender’s influence on subjective age is crucial to better understanding gender differences in older adults’ well-being. In our symposium we bring gender to the center of subjective age scholarship. Barrett, Michael, & Noblitt begin by establishing that subjective age research should portray gender as a social-level, rather than individual-level, characteristic. As a complement, Turner, Settersten, and Hooker illustrate how gender has or has not been included in the four theoretical domains of subjective age (self-perceptions of aging, old age stereotypes, age identity, and awareness of age related change), and offer insights into how gender might be included in future studies on each domain. We then shift to two papers presenting new empirical analyses on the role gender plays in subjective age. Kornadt shares how men and women’s commitment to certain social roles differentially informs their subjective age, while Settersten, Day, and Hagestad turn attention to a double standard of aging for women and men with evidence across Europe. Discussant Toni Calasanti closes by offering thoughts on the future of subjective age and gender scholarship, including considering gender beyond the binary.

